# Biooxidation of Iron by *Acidithiobacillus ferrooxidans* in the Presence of D-Galactose: Understanding Its Influence on the Production of EPS and Cell Tolerance to High Concentrations of Iron

**DOI:** 10.3389/fmicb.2020.00759

**Published:** 2020-04-23

**Authors:** Albert Saavedra, Paulina Aguirre, Juan Carlos Gentina

**Affiliations:** ^1^Escuela de Ingeniería Bioquímica, Pontificia Universidad Católica de Valparaíso, Valparaíso, Chile; ^2^Departamento de Química y Ciencias Exactas (Sección de Ingeniería Ambiental), Universidad Técnica Particular de Loja, Loja, Ecuador

**Keywords:** *At. ferrooxidans*, iron biooxidation, extracellular polymeric substances, D-galactose, tolerance to ferric ion

## Abstract

*Acidithiobacillus ferrooxidans*, together with other microorganisms, has an important role on biohydrometallurgical processes. Such bacterium gets its energy from the oxidation of ferrous ion and reduced sulfur; in the first case, the accumulation of ferric ion as a product can cause its inhibition. It is known that the extracellular polymeric substances (EPS) may have an important role in the adaptation and tolerance to diverse inhibiting conditions. In the present study, it was tested how D-galactose can influence the production of extracellular polymeric substances (EPS) on *At. ferrooxidans* by evaluating at the same time its biooxidant activity and capacity to tolerate high concentrations of ferric ion. The visualization and quantification of EPS was done through a confocal laser scanning microscope (CLSM). The results show that at low cellular concentrations, the D-galactose inhibits the microbial growth and the biooxidation of ferrous ion; however, when the quantity of microorganisms is high enough, the inhibition is not present. By means of chemostat tests, several concentrations of D-galactose (0; 0.15; 0.25; and 0.35%) were evaluated, thus reaching the highest production of EPS when using 0.35% of this sugar. In cultures with such concentration of D-galactose, the tolerance of the bacterium was tested at high concentrations of ferric ion and it was compared with cultures in which sugar was not added. The results show that cultures with D-galactose reached a higher tolerance to ferric ion (48.15 ± 1.9 g L^–1^) compare to cultures without adding D-galactose (38.7 ± 0.47 g L^–1^ ferric ion). Also it was observed a higher amount of EPS on cells growing in the presence of D-galactose suggesting its influence on the greater tolerance of *At. ferrooxidans* to ferric ion. Therefore, according to the results, the bases of a strategy are considered to overproduce EPS by means of *At. ferrooxidans* in planktonic state, so that, it can be used as a pre-treatment to increase its resistance and tolerance to high concentrations of ferric ion and improve the efficiency of *At. ferrooxidans* when acting in biohydrometallurgical processes.

## Graphical Abstract

Planktonic cells of *At. ferrooxidans* produce EPS only when grown in the presence of D-galactose as can be seen in the CLSM images. Cells having EPS on their surface can tolerate higher iron concentrations compare to cells without a layer of EPS surrounding them.

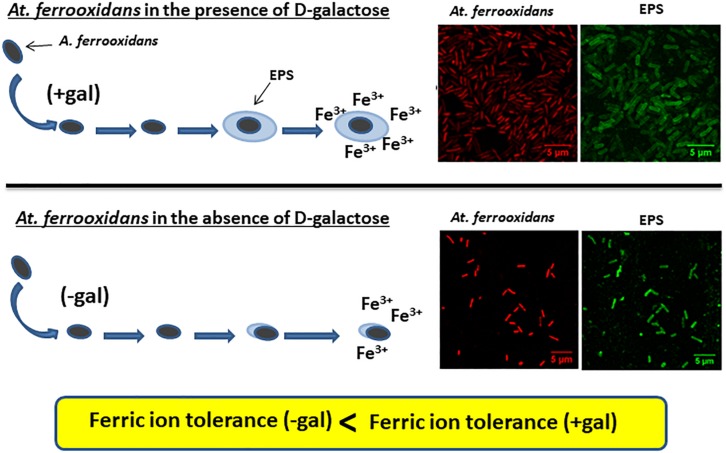

## Introduction

*Acidithiobacillus ferrooxidans* is an acidophilic and chemolithoautotrophic bacterium that obtains its energy from the oxidation of ferrous ion and reduced sulfur compounds; and as a carbon source, it uses CO_2_ (Kelly and Wood, 2000). This microorganism is common in biotechnological processes such as bioleaching and biooxidation of minerals, in which there is accumulation of ferric ions (Fe^3+^) that results from the energy obtaining reactions of the cell. *At. ferrooxidans* can be inhibited by ferrous (Fe^2+^) and ferric (Fe^3+^) ions, being stronger the last one. Thus for example, it has been reported that for ferrous ion and for ferric ion the concentrations causing inhibition are greater than 30 and 20 g L^–1^, respectively (Lizama and Suzuki, 1989; Boon et al., 1999). It is known that Fe^3+^ inhibits microbial growth since it acts according to the mechanisms of competitive and non-competitive product inhibition (Nemati and Harrison, 2000) and causes low process productivity (Kawabe et al., 2003).

In recent studies, it has been reported that the use of monosaccharides induces the production of extracellular polymeric substances (EPS) in chemolithoautotrophic microorganisms (Bellenberg et al., 2012) without causing inhibition of microbial growth when they are sufficiently adapted to the substances (Aguirre et al., 2018). Thus, Barreto et al. (2005b) identified the functional presence of gal operon in *At. ferrooxidans*, which is mostly expressed in cells that have iron as an energy substrate in relation to those that have sulfur, and three precursors related to EPS biosynthesis in *At. ferrooxidans* − UDP-glucose, UDP-galactose, and dTDP-rhamnose - all of them synthesized from glucose-1-phosphate. Also, Barreto et al. (2005a), through bioinformatics studies, identified monosaccharide transporting proteins in *At. ferrooxidans* associated to the production of EPS, suggesting that D-galactose may fulfill the role of inducer in the formation of EPS on sessile bacteria.

Bellenberg et al. (2012) also reported increased biofilm formation on mineral in the presence of D-galactose and D-glucose and recently, Aguirre et al. (2018) showed that adding D-galactose in a culture of *L. ferrooxidans* increased the production of EPS. Apparently, the presence of these carbohydrates in small concentrations could be stimulating the production of EPS on planktonic and sessile cells.

Extracellular polymeric substances are one of the main components of biofilms. They are formed of proteins, sugars, lipids, nucleic acids and cell fragments whose composition and concentration may vary depending on microorganisms and environmental conditions (Wingender et al., 1999). The EPS fulfill different functions: reaction space for oxidation, forming an active and absorbing surface, favoring bacterial adhesion, helping the structural stabilization of the biofilm and forming a protective layer against biocides or other media inhibitors, among others (Wingender et al., 1999; Michel et al., 2009). EPS origin is mainly due to secretion processes and cell lysis, thus being accumulated on the surfaces of cells, or disseminated in the medium (Wingender et al., 1999). The production of EPS in microorganisms is usually a cellular response to harsh environmental conditions such as extreme temperatures, presence of metal ions, inhibitors, among others (Wenbin et al., 2011; Yu et al., 2013) and also, its production is one of the strategies in the process of adaptation of microorganisms (Geyik and Çeçen, 2015). The chemical composition of EPS changes depending on the physical-chemical conditions of the environment, reporting different compositions exuded by *At. ferrooxidans* when faced with pyrite, elemental sulfur or iron (Gehrke et al., 1998).

The presence of EPS in biohydrometallurgical processes would favor bacterial attachment to the mineral and its subsequent role in the formation of biofilm on minerals, increasing the chemical attack on the mineral, and therefore the efficiency of the process. Hence, the production of EPS in planktonic cells can generate cells with high attachment capacity (Saavedra et al., 2018) in addition to conferring protection against harsh conditions (Wenbin et al., 2011). There are studies where different processes have been carried out for the generation of EPS in planktonic cells; for example, Biosigma, a biomining development company, presented a patent where a strategy to increase the production of EPS is described; thus, by silencing genes through redirecting the source of carbon to the formation of EPS and not to the formation of biomass (Mass et al., 2015). Therefore, it is important to address the search for new strategies to increase the production of EPS in bacteria participating in biomining processes. In this regard, it is also necessary to know the role of microorganisms in the formation of EPS and their interaction among them, as in the case of *L. ferrooxidans* and *At*. *thiooxidans* that colonize a mineral faster when it is pre-colonized by *At. ferrooxidans* (Bellenberg et al., 2014); or the case of *At. ferrooxidans* which form biofilm on mineral substrates faster in the presence of *Acidiphilium* sp. thus obtaining a greater solubilization of the mineral (Yang et al., 2013).

It has been proven in other microorganisms that the presence of EPS increases resistance and tolerance to metal ions or other toxic agents (van Acker and Coenye, 2016). Yu et al. (2013) showed that, when EPS increases in *At. ferrooxidans*, it has a higher adsorption capacity of iron and copper; and therefore greater tolerance. Also, with other bacteria, it has been shown that EPS can increase tolerance to nanoparticles (Ouyang et al., 2017) and mercury (Cruz et al., 2017). The chemical or physicochemical interaction (adsorption) of EPS with metal ions and toxic compounds increases bacterial tolerance against toxic agents.

In this same sense, Tapia et al. (2013) indicate that the EPS exuded from *At. ferrooxidans* has the ability to associate with Fe^2+^ and Fe^3+^ ions. The binding of iron happens especially on the carboxyl groups of EPS forming complex oxalates of Fe^2+^ (FeC_2_O_4_) and Fe^3+^ (K_3_FeC_6_O_12_⋅3H_2_O) and other organic substances such as iron formate (C_3_H_3_FeO_6_⋅H_2_O); accumulating in the EPS 516.7 ± 23.4 mg Fe/g EPS approximately at an initial metal concentration of 2.0 g L^–1^ of Fe_Total_. Although the EPS does not represent a greater capacity for iron oxidation, a greater accumulation of Fe^3+^ can increase the bioleaching rate in minerals.

Therefore, the present work focuses on the evaluation of ferrous iron oxidation under conditions of EPS over production induced by D-galactose in planktonic cells of *At. ferrooxidans* at high ferric iron concentrations. The results of this study could be used as the basis for applying strategies to obtain planktonic microbial inoculums containing EPS and high capacity for ferric ion tolerance to be applied in heap and bioreactors, shifting the balance toward a greater number of cells attached to the particles, which would mean higher speed of solubilization.

## Methodology

### Microorganism and Culture Medium

*Acidithiobacillus ferrooxidans* DSMZ 11477 was used throughout this study. The cells were cultured in Kim medium (g L^–1^): KCl, 0.1; MgSO_4_⋅7H_2_O, 0.2; (NH_4_)H_2_PO_4_, 2.0; and supplemented with FeSO_4_⋅7H_2_O, 44; the pH was adjusted to 1.8 with H_2_SO_4_ 10N (Kim et al., 2002). For cultures at other iron concentrations, the supplemented FeSO_4_⋅7H_2_O was varied; while, the other salts of the culture medium were maintained.

### Evaluation of EPS Production by Adding D-Galactose

Microbial growth and iron biooxidation assays were carried out in batch cultures to determine how D-galactose can influence the growth of *At. ferrooxidans*. The tests were performed in 500 mL Erlenmeyer flasks with a culture volume of 200 mL. Kim culture medium enriched with 0.25% D-galactose was used. The pre-inoculum concentration was 10^6^ cells mL^–1^. The flasks were incubated at 30°C and 200 rpm in a rotary incubator.

Reactor tests were performed, operating under the continuous culture mode (simple chemostat), to evaluate the production of EPS in *At. ferrooxidans*. In this modality, it was operated using Kim medium adding different concentrations of the D-galactose as inducer in the feed (0, 0.15, 0.25, and 0.35%).

The initial inoculum to carry out the experiments in the reactor was grown at 30°C, 200 rpm in 500 mL flasks with 100 mL of Kim medium supplemented with 44 g L^–1^ ferrous sulfate. The experiments were performed in a 1 L reactor with a working volume of 0.5 L, pH controlled at 1.8 by automatic addition of 10 N H_2_SO_4_ and a constant temperature of 30°C. The stirring was adjusted at 200 rpm, and the air flow to the reactor was 0.5 L min^–1^.

The chemostat operation was preceded by a batch culture. When ferrous ion (Fe^2+^) had been consumed about 3/4 of its initial concentration, the discontinuous mode was changed to continuous at a dilution rate (D) of 0.03 h^–1^. It was considered that the steady state had been reached after at least three hydraulic residence times (τ) with a variation in cell concentration between samples less than 10%. Four disturbances were made to the system and different stationary states were obtained which varied according to D-galactose feed concentrations (0.15, 0.25, and 0.35%). The samples were made in triplicate and the results are presented as averages and mean deviations.

### Tolerance Tests to Ferric Ion (Fe^3+^)

Tolerance tests were performed in the absence and presence of D-galactose. The tolerance of *At. ferrooxidans* to ferric ion was determined in batch cultures. Cells were cultured in 500 mL Kim medium in a reactor of 1000 mL, at 30°C, initial pH of 1.8, 200 rpm and inoculum of 10% (v/v). The high concentrations of ferric ion were obtained by accumulation after adding consecutively a pulse equivalent to 9 g L^–1^ of ferrous ion after consumption in the previous culture stage, until observing a total inhibition of cells growth and ferrous ion oxidation. In the test with presence of D-galactose, the sugar was added with the first pulse of ferrous ion (second stage). The assays were done in triplicate. At each stage the volumetric productivity of ferric ion (${\text{Q}}_{{\text{Fe}}^{3+}}$) and the accumulated specific productivity of ferric ion (${\bar{\text{q}}}_{{\text{Fe}}^{3+}}$), were calculated.

(1) $${\text{Q}}_{{\text{Fe}}^{3+}}=\frac{\Delta \,\left[{\text{Fe}}^{3+}\right]}{\Delta \,\text{t}}$$

(2) $${\bar{\text{q}}}_{{\text{Fe}}^{3+}}=\frac{\Delta \,\left[{\text{Fe}}^{3+}\right]}{\Delta \left[\mathrm{No}.\mathrm{cells}\right]\times \Delta \text{t}}$$

Where: [Fe^3+^] is the ferric ion concentration (g L^–1^); t is the time (h); [No. cells] is the cell concentration (cells L^–1^); and Δ represents the increment of the variable at each stage.

### Bacteria and EPS Visualization and Volume Quantification

The EPS were visualized using a confocal laser scanning microscope (CLSM). For each analysis, 1 mL of sample was taken and preserved with 2% glycerol at 5°C until processing. 10 μL of samples were fixed in glass slide during 10 min and then immersed in methanol for 10 min. Two fluorophores were used, labeling the cells nucleic acids with propidium iodide (PI) and EPS carbohydrates with wheat germ agglutinin (WGA). Each fluorophore was incubated with the sample for 2 h and visualized under appropriate laser excitation wavelengths and optical filters for specific detection of fluorophore signals (argon laser: 488 nm, 505–550 nm bandpass filter; helium neon laser: 543 and >560 nm longpass filter). The EPS volume was measured using ImageJ, an image processing software, allowing to quantify bio-volumes (μm^3^) and relative fluorescence of cells and EPS.

### Analytical Methods

The total iron and ferrous iron were measured using the 1,10-phenanthroline colorimetric method; ferric ion was determined by the difference between the two values, as described previously (Muir and Andersen, 1977). The microbial concentration was determined by Petroff Hausser counting chamber, and the pH and ORP were measured with commercial electrodes.

## Results

### Effect of D-Galactose Addition on Iron Biooxidation by *At. ferrooxidans*

**FIGURE 1:**
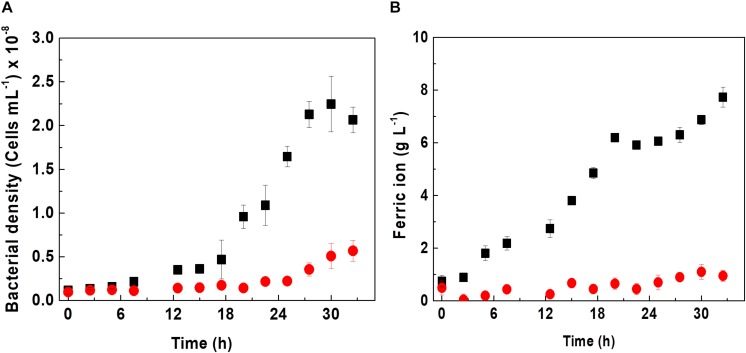
Effect of D-galactose on growth **(A)** and biooxidation of iron **(B)** in a culture of *At. ferrooxidans*. Curves indicate absence (■) and presence (•) of 0.25% of D-galactose. The vertical bars correspond to the standard deviation (*n* = 3).

Since D-galactose is not a substrate used in cultures of *At. ferrooxidans*, bacterial growth and iron oxidation kinetics were evaluated in the presence and absence of 0.25% D-galactose as shown in Figure 1.

*Acidithiobacillus ferrooxidans* in the absence of D-galactose exhibited a specific maximum growth rate (μ_max_) of 0.11 h^–1^ in Kim medium; however, in the presence of D-galactose, it showed a lower μ_max_ of 0.009 h^–1^ as can be appreciated from Figure 1A. Likewise, it is verified that the cell growth without D-galactose reached a cell concentration of approximately 2.2 × 10^8^ cells mL^–1^; while those microorganisms subjected to D-galactose, barely reached 0.5 × 10^8^ cells mL^–1^. According to Tuovinen and Kelly (1973), D-galactose can exert an inhibitory effect on the growth of *At. ferrooxidans* in an *in vitro* culture if these microorganisms are not previously adapted to this substance. However, it has been reported that in mining environments *At*. *ferrooxidans* is no stranger to the presence of carbohydrates. This is due to the presence of microbial consortia (heterotrophs and autotrophs), including bioleaching microorganisms (Bhattacharyya et al., 1991; Jia et al., 2019), which exude EPS in either planktonic or sessile state, releasing carbohydrates into the medium, including glucuronic acid (oxidized form of D-galactose) and glucose among its components, as reported by Gehrke et al. (1998), for the case of *At. ferrooxidans*. It may be the origin of organic components in mining environments and also from the oxidation of cell debris. In this regard, Liu et al. (2011) managed to obtain an increase in the efficiency of the bioleaching process, occurring when these two bacteria grew together, possibly due to a synergism in the production of carbohydrates by *Acidiphilium sp.* and the production of EPS by *At. ferrooxidans*. In this same sense, it has been reported that adding carbohydrates such as galactose or glucose to a culture medium with a high concentration of *At*. *ferrooxidans* and in the presence of mineral increases the rate of biofilm formation (Barreto et al., 2005a; Bellenberg et al., 2012). The results of biooxidation activity shown in Figure 1B are in agreement with the behavior of *At. ferrooxidans* observed in Figure 1A.

**FIGURE 2:**
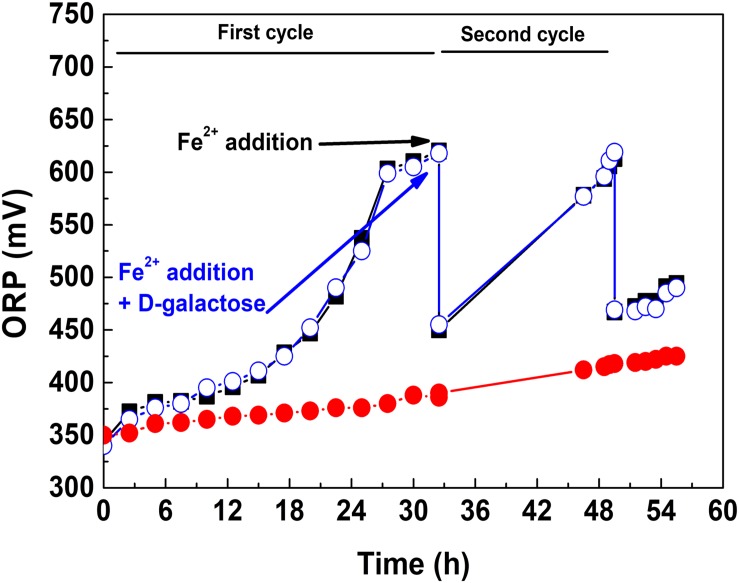
ORP variation in the presence and absence of D-galactose: D-galactose added from the beginning of the culture (•), D-galactose not added and two cycle culture (■), D-galactose added at the beginning of second cycle culture (∘).

Considering the aforementioned, it was taken the strategy of adding D-galactose once the microbial population reached a high concentration. This is shown in Figure 2, where three culture conditions are presented. In the first, D-galactose was added at the beginning of the culture. The redox potential (ORP) increased around 50 mV, indicative of a low oxidation of ferrous iron. In the second, D-galactose was not added and adding the equivalent of 9 g L^–1^ of ferrous iron performed a second growth cycle. It was observed a typical oxidation behavior, reaching ORP close to 625 mV in both cycles. The fall of ORP between cycles was due to the change of the Fe^3+^/Fe^2+^ ratio. In the third, the culture started like the second one, but 0.25% of D-galactose was added at the beginning of the second cycle. The behavior of ORP from that point in the second and third cultures was quite similar, indicating that there is no inhibition in the biooxidative process of Fe^2+^ in the presence of D-galactose. This test demonstrated that *At. ferrooxidans* in the presence of D-galactose exhibits inhibition at low cell concentration and that it can be reversed at high cell concentration.

In this same sense, it has been shown that a high concentration of cells can overcome adverse conditions, probably because it encourages the cellular communication (Atkinson and Williams, 2009; Gao et al., 2020). *At. ferrooxidans* presents quorum sensing of type I (Zhang et al., 2018), and this communication allows cell-cell signaling through self-inducing molecules, allowing the regulation of different cell processes that are dependent on the density of the microbial population, including EPS production, adaptation and tolerance (Gao et al., 2020).

### Determination of the Condition for the Production of EPS by *At. ferrooxidans*

**FIGURE 3:**
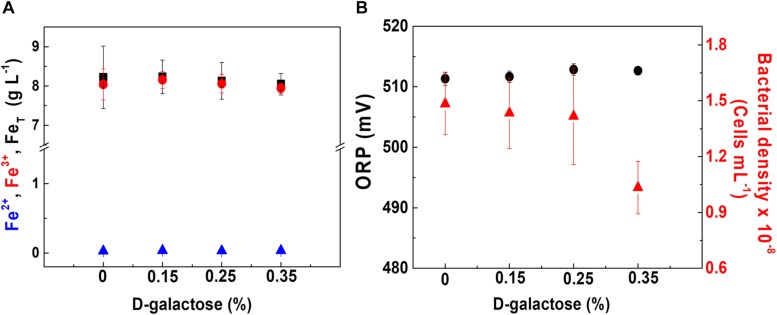
*At. ferrooxidans* culture behavior at different concentrations of D-galactose. **(A)** Concentration of total iron (■), ferric ion (•), and ferrous ion (▲). **(B)** ORP (•) and bacterial density (▲). Chemostat operated at *D* = 0.03 h^– 1^, pH 1.8, and 30°C. Samples were taken at steady state conditions.

Considering that one of the advantages of using chemostat is the evaluation of variables maintaining the constant metabolic state of microorganisms, the strategy of assessing the effect of D-galactose on oxidation and bacterial growth was carried out. The test was performed at a dilution rate (D) of 0.03 h^–1^, low enough in order to obtain a higher cell concentration considering the result shown in Figure 2. Therefore, for this test, each one of the concentrations of D-galactose (0.15, 0.25 and 0.35% w/v) was evaluated. The behaviors of iron concentration, bacterial density and ORP in each of the stationary states are shown in Figure 3.

In Figure 3A, it is observed that at the different concentrations of D-galactose tested, iron concentration has a similar behavior, indicating that there is no inhibitory effect on the biooxidation activity of *At. ferrooxidans*. In Figure 3B, the ORP and bacterial density values are kept constant for D-galactose concentrations of 0.15 and 0.25%. However, at 0.35% there was a decrease in bacterial density, possibly due to a phenomenon of inhibition by the sugar on microbial growth, nevertheless its biooxidation activity was not modified. The latter may be due to the activation of metabolic pathways that favor biooxidation (Ruiz et al., 2008; Vu et al., 2009) and a possible redistribution of the energy obtained by *At. ferrooxidans* through ferrous ion oxidation, with respect to its use in the formation of biomass, some products and cell maintenance (Pirt, 1965).

**FIGURE 4:**
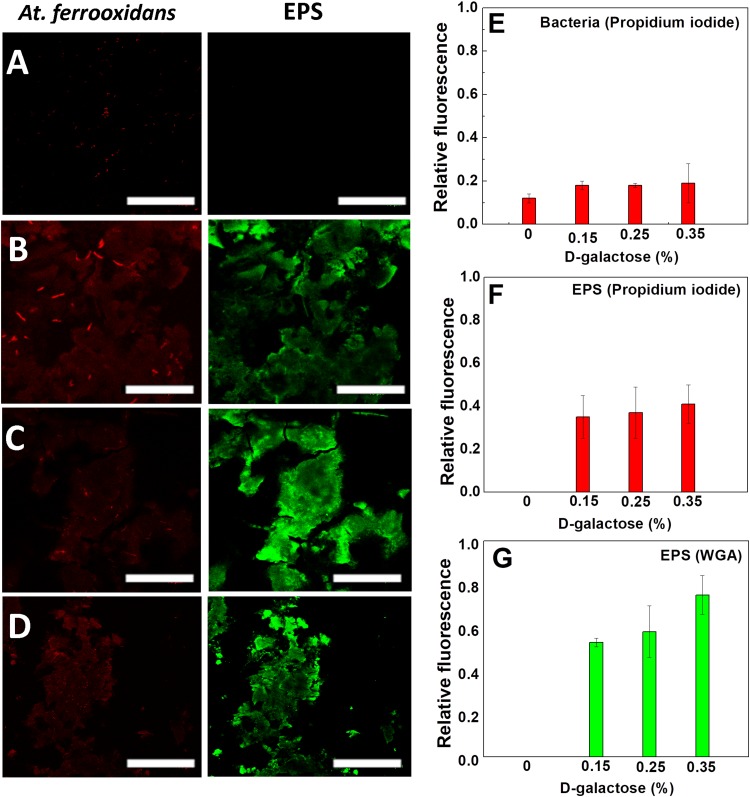
Images of CLSM of *At. ferrooxidans*. Bacteria, marked with propidium iodide, are observed (red) and EPS marked with WGA (green) in each stationary state obtained from continuous culture. D-galactose **(A)** 0%, **(B)** 0.15%, **(C)** 0.25%, **(D)** 0.35%. The bar corresponds to 10 μm. Relative fluorescence of *At. ferrooxidans* **(E)** and EPS **(F,G)** obtained from CLSM images.

In Figure 4, the CLSM images are shown in which the carbohydrates of EPS and nucleic acids of *At. ferrooxidans* are marked. The images come from samples obtained after reaching the steady state of the chemostat at different concentrations of D-galactose (0, 0.15, 0.25, and 0.35% w/v).

As it is seen in Figure 4A, in the absence of D-galactose, it was not possible to visualize EPS unlike samples that were subjected to different concentrations of D-galactose. The conditions, in which generation of EPS was observed, were in the samples subjected to 0.15, 0.25 and 0.35% of D-galactose (Figures 4B–D). Although it is true that in each of the stationary states the iron oxidation was similar, the amount of EPS produced by the bacteria was different. This information was verified with the determination of the relative fluorescence to samples obtained at different concentrations of D-galactose. Figure 4G shows that the highest production of EPS occurs with the condition of 0.35% D-galactose. A similar study in *Leptospirillum ferrooxidans*, where it was tested with 0.15%, 0.25% and 0.35% of D-galactose, also showed a higher production of EPS while increasing D-galactose (Aguirre et al., 2018).

Figure 4E shows that the fluorescence of *At. ferrooxidans* slightly increased in the presence of D-galactose. Figures 4F,G show the relative fluorescence of the EPS generated by *At. ferrooxidans*, where it is noted that propidium iodide also marks the EPS (Figures 4B–D), probably due to the presence of nucleic acids forming the EPS. The presence of nucleic acids in EPS has been discussed by different authors; studies related to biofilm of *Pseudomonas aeruginosa* indicate that the nucleic acids in the EPS have an intercellular connector function (Allesen-Holm et al., 2006; Flemming et al., 2007; Yang et al., 2007). Also, studies by Gehrke et al. (1998), Kinzler et al. (2003) reported that the composition of EPS may vary depending on the presence of sulfur, iron and mineral substrate. The presence of nucleic acids in EPS, expelled as RNA messenger by the bacteria, has been observed in other microorganisms (Tahrioui et al., 2019).

### Tolerance Tests to High Concentrations of Ferric Ion (Fe ^3+^)

Tolerance tests to high concentrations of ferric ion were carried out in the absence and presence of 0.35% D-galactose (best condition obtained in the previous section). The experience was based on the addition of consecutive pulses of equivalent 9 g L^–1^ of ferrous ion after consumption in the previous stage, until the biooxidation activity of *At. ferrooxidans* stopped completely. This strategy allowed the progressive increase of ferric ion concentration, eliminating the possible inhibition by ferrous ion.

The maximum tolerance of *At. ferrooxidans* to ferric ion in the absence of D-galactose was 38.7 ± 0.47 g L^–1^, and in the presence of 0.35% D-galactose was 48.15 ± 1.9 g L^–1^. The tolerance of *At. ferrooxidans* to ferric ion has been addressed by different researchers, reporting ferric ion tolerance concentrations lower than those found in this research, as in the case of Lizama and Suzuki (1989), Muzzio (2002), Kawabe et al. (2003), who reported that concentrations of 15, 18.3, and 20 g L^–1^ of exogenous ferric ion, respectively, exert a total inhibition on *At. ferrooxidans*.

**FIGURE 5:**
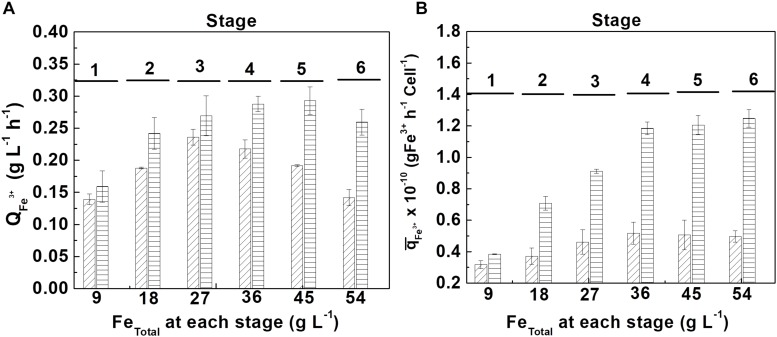
Tolerance evaluation of *At. ferrooxidans* to high concentrations of accumulated ferric ion by adding ferrous ion pulses. **(A)** volumetric productivity (${\text{Q}}_{{\text{Fe}}^{3+}}$) and **(B)** accumulated specific productivity (${\bar{\text{q}}}_{{\text{Fe}}^{3+}}$) at each ferrous ion pulse in the absence (diagonal lines) and in the presence (horizontal lines) of D-galactose. (Samples number = 3). The length of time of each stage is show in Supplementary Table S1 (Supplementary Material).

At the tolerance tests, the volumetric productivity (${\text{Q}}_{{\text{Fe}}^{3+}}$) and the accumulated specific productivity (${\bar{\text{q}}}_{{\text{Fe}}^{3+}}$) of ferric ion were calculated with respect to the biomass produced, for each stage, which are shown in Figure 5.

From Figure 5A, it can be seen that the bacteria growing in the presence of 0.35% of D-galactose presented a higher volumetric productivity than the bacteria cultured in the absence of the sugar. In the first case the maximum productivity was obtained at the fifth stage (45 g L^–1^ of total iron) whereas in the second case it was only at the third stage (27 g L^–1^ of total iron). The better performance of the cells cultured in the presence of D-galactose is confirmed in Figure 5B that shows a marked difference of the specific biooxidation activity of *At. ferrooxidans* according to the presence or absence of the sugar in the culture medium.

**FIGURE 6:**
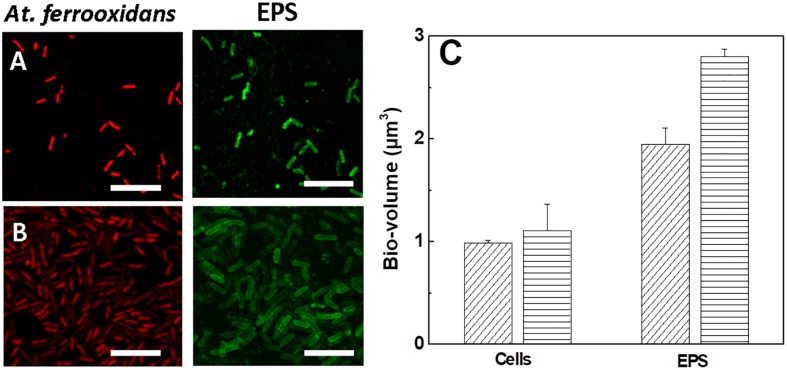
Images and bio-volumes of cells and EPS of *At. ferrooxidans* grown in the absence and presence of D-galactose standing maximum tolerance to ferric ion. **(A)** Images of CLSM of *At. ferrooxidans* in the absence of D-galactose and **(B)** in the presence of 0.35% of D-galactose. **(C)** Bio-volume of bacteria and EPS for individual cell obtained from images **(A,B)** in the absence (diagonal lines) and presence (horizontal lines) of D-galactose. The bar corresponds to 5 μm.

Samples of *At. ferrooxidans* standing the respective maximum tolerance concentration of ferric ion cultivated in the presence and absence of D-galactose were analyzed by CLSM, as shown in Figure 6.

In general Figures 6A,B show the production of EPS in bacteria exposed to high concentrations of ferric ion. The EPS is observed individually, surrounding each cell, apparently in a homogeneous distribution. A higher volume of EPS is visualized in bacteria that were exposed to 0.35% D-galactose. This is corroborated by quantifying the bio-volume of the EPS (Figure 6C) where the cells cultured in the presence of 0.35% D-galactose has around 25% more EPS than cells cultured without D-galactose. In addition, it was observed that bacteria were larger than usual. Previous studies have reported the increase of bacterial EPS in stress situations, such as being subjected to high concentrations of copper (Yu et al., 2013). On the other hand, it is known that bacteria show morphological changes when they are in stressful situations (Young, 2006; Yu et al., 2013). This change in size of *At. ferrooxidans* was also observed by Barreto et al. (2005a) who attributed it to the overproduction of EPS.

## Conclusion

In the range studied of D-galactose addition to the culture medium, the highest volume of EPS produced by *At. ferrooxidans* was obtained when using 0.35% of D-galactose. When no D-galactose was added, EPS formation was not observed.

The maximum tolerance to ferric ion concentration and consequent biooxidation activity of *At. ferrooxidans* was 24% higher for cells grown in the presence of 0.35% of D-galactose than those grown in the absence of the sugar. The cells with the higher maximum tolerance also had an EPS bio-volume 25% higher, correlating cellular EPS quantity with maximum tolerance to ferric ion concentration.

The inhibitory effect of D-galactose on *At. ferrooxidans* growth was overcome when adding the sugar to a culture of active cells amounting high cell density.

The presence of D-galactose in the culture medium increased the production and accumulation of EPS by *At. ferrooxidans*, being a determining factor for tolerance to high ferric ion concentrations.

This strategy could also be used (individually and in consortia) with other bacteria related to biohydrometallurgical processes such as *L. ferrooxidans*, *L. ferriphillum* and *At. thiooxidans* in order to understand its applicability in biomining processes.

## Data Availability Statement

The datasets generated for this study are available on request to the corresponding author.

## Author Contributions

JG proposed and designed the idea and the study, provided the facilities, funding, revised and commented, and contributed to writing of the manuscript. AS proposed and designed the idea and the study, performed the experiments, collected, processed and analyzed data, was involved in the study design, and contributed to writing of the manuscript. PA was involved in data analysis, discussed the results, and contributed to writing of the manuscript.

## Conflict of Interest

The authors declare that the research was conducted in the absence of any commercial or financial relationships that could be construed as a potential conflict of interest.
