# Supplementary material for: Biooxidation of Iron by Acidithiobacillus ferrooxidans in the Presence of D-Galactose: Understanding Its Influence on the Production of EPS and Cell Tolerance to High Concentrations of Iron
Source: Front Microbiol. 2020 Apr 23;11:759. doi: 10.3389/fmicb.2020.00759 (PMC7191041; doi:10.3389/fmicb.2020.00759)
Supplement: Supplementary file 1 [file Data_Sheet_1.docx]

Supplementary Material

Biooxidation of iron by *Acidithiobacillus ferrooxidans* in the presence of D-galactose: Understanding its influence on the production of EPS and cell tolerance to high concentrations of iron

**Albert Saavedra^1^, Paulina Aguirre^1,2^, Juan Carlos Gentina^1*^**

^1^Escuela de Ingeniería Bioquímica, Pontificia Universidad Católica de Valparaíso, Avenida Brasil 2085, Valparaíso, Chile.

^2^Universidad Técnica Particular de Loja (UTPL), Loja, Ecuador.

*** Correspondence:**Corresponding Author
carlos.gentina@pucv.cl (J.C. Gentina).

**Table S1. Length of time of each stage in Figure 5**

| **Stage** | **Total iron (g L^-1^)** | **Length of time (h)** | |
| --- | --- | --- | --- |
|  |  | **With D-galactose** | **Without D-galactose** |
| 1 | 0 to 9 | 38.2 | 40.5 |
| 2 | 9 to 18 | 34.4 | 47.8 |
| 3 | 18 to 27 | 33.8 | 36.7 |
| 4 | 27 to 36 | 35.1 | 36.5 |
| 5 | 36 to 45 | 40.3 | 47.4 |
| 6 | 45 to 54 | 42.1 | 57.9 |
